# Supplementary material for: The Longevity of Colonies of Fungus-Growing Termites and the Stability of the Symbiosis
Source: Insects. 2020 Aug 13;11(8):527. doi: 10.3390/insects11080527 (PMC7469218; doi:10.3390/insects11080527)
Supplement: Supplementary file 1 [file insects-11-00527-s001.pdf]

Table S1. Open questions and possible approaches to answer these questions. Grouped by subject.

Subject	Open Questions	Possible Approaches
**The Stability of the Termite Mound**		
	Is an abandoned eroding mound perceived as a good site to colonise?	Field study: Observe abandoned mounds and how often they are colonized compared to new sites.
	Is there higher survival of new colonies in existing mounds?	Field study: Observe colonized abandoned mounds and score the survival of the colony compared to the survival at new sites.
	What is the maximum lifespan of reproductives of colonies in different species of fungus-growing termites?	Field study: Long-term observation of different termite species with the frequent observation of the survival of marked 1eproductive.
**How Short-Term Stability of the Symbiont May Affect Long-Term Stability of the Symbiosis**		
Symbiont transmission and dispersal	What is the mechanism behind the timing between fruiting and the appearance of new workers?	Field experiment/lab colonies: Change conditions by removing termites, changing temperature or humidity level, observe if these factors affect fungal fruiting.
Selection of the "right" partner	How does specificity arise and how do certain host-symbiont combinations arise?	Comparative phylogenetic analysis: Study the correlation of specific species combinations with specific environmental factors.
	Do environmental factors explain variation in fungal symbionts between mounds of the same termite species?	Field observations combined with comparative analysis: Measure environmental factors and study correlations between these factors and fungal symbionts.
	To what extent can the termite control environmental factors of a colony, and what is the flexibility within species?	Field observations: Measure environmental factors (e.g., temperature, moisture level and construction of the termite mound), and compare different termite colonies.
	Do termite species show flexibility in the way they build their mounds?	Field observations: Study fungal mounds of the same termite species at different sites.

	Do environmental factors affect the establishment of a particular fungal symbiont?	Field observations combined with field experiments/lab colonies: Study correlations between environmental factors and certain fungal symbionts, and manipulate environmental factors to see if this affects the establishment of fungal species.
	How is the growth of *Termitomyces* affected by different environmental factors?	Lab experiment: Cultivate fungal species on different substrates and environmental conditions and measure growth.
	Can the fungus trigger termite behaviors to change the environmental conditions?	Field experiment/lab colonies: Manipulate fungal growth and measure termite behavior, rear termite species with different fungi.
	How many *Termitomyces* species are brought into the primordial comb by the first termite workers?	Field observations/lab colonies and genetic analysis: Sample fungi right after the establishment of a new termite colony, genetically compare the different samples to study genetic variation.
	Is there competition between different *Termitomyces* species in the primordial comb?	Field observations/lab colonies and genetic analysis: Continue sampling after the establishment of a colony and study changes in genetic frequency.
	Do the termites actively select *Termitomyces* species, or is this a passive process?	Field experiment/lab colonies: Expose termites to different *Termitomyces* species all equally well adapted to that environment in different frequencies and observe if the termites select and maintain their adapted *Termitomyces* species in all frequencies.
	Do termites react differently to different *Termitomyces* species?	Field experiment/lab colonies: Expose termites to different *Termitomyces* species and observe their behavior.
	Is there any active selection of a specific fungal strain, or is the whole process passive and just depending on density and performance of the fungus?	Field experiment/lab colonies: Expose termites to different *Termitomyces* strains all equally well adapted to that environment in different frequencies and observe if the termites select and maintain a specific *Termitomyces* strain in all frequencies.
Conflict Reduction between Termite Host and Fungal Symbiont	How do the termites suppress fruiting of their fungal symbiont?	Field experiments and observations/lab colonies: Exclude termites from certain fungal combs, and observe fungal growth, observe termite behavior around fungi.
	How does the fungus influence the reproduction of termites?	RNA expression analysis: Measure if expression levels change when termites reproduce.
	Has *Termitomyces* evolved mechanisms to influence termite behavior?	Genetic analysis: Comparison with free-living relatives and scan for genes which might cause the expression of exudates which might affect termites.

Conflict between Symbionts: Establishment and Maintenance of Fungal Monocultures	How is the monoculture established in primordial combs, which selection pressures play a role?	Field observations/lab colonies and genetic analysis: Continue sampling after the establishment of a colony and study changes in genetic frequency. Alternatively, controlled experiments with lab colonies.
	How long does it take to form a monoculture?	Field observations/lab colonies and genetic analysis: Continue sampling after the establishment of a colony and study changes in genetic frequency.
	How long does it take to select the dominant heterokaryon and to eliminate the less frequent heterokaryons?	Field observations/lab colonies and genetic analysis: Continue sampling after the establishment of a colony and study changes in genetic frequency.
	Does fungal turnover occur over time?	Long-term field observations/lab colonies and genetic analysis: Continue sampling after the establishment of a colony and study changes in genetic frequency; expose termites in the lab to spores of different *Termitomyces* species.
	Which mechanisms underlie the maintenance of a monoculture? Only positive frequency-dependent selection or also other mechanisms?	Field observations/lab colonies: Continue studying the termite behavior after the establishment of a colony.
	How does the termite prevent cheating nuclei or cells from spreading?	Field observations and experiments/lab colonies: Continue studying the termite behavior after the establishment of a colony and introduce cheating mutants to the fungal comb to observe how termites react to such mutants.
	Are termites able to discriminate between cooperators and cheaters?	Field experiments/lab colonies: Introduce cheating mutants to the fungal comb to observe how termites react to such mutants.
	Do the termites have mechanisms to prevent the spread of cheaters?	Field experiments/lab colonies: Introduce cheating mutants to the fungal comb to observe how termites react to such mutants.
	Is the mutation rate of *Termitomyces* reduced, and how?	Lab experiments: Long-term evolution experiment to sequentially transfer fungi, study the accumulation of mutations.
	Do the termites carefully monitor the fungi for mutations?	Field experiments/lab colonies: Introduce cheating mutants to the fungal comb to observe how termites react to such mutants.
